# Supplementary material for: Pension levels of chinese institutions during the transition period: A case study of universities
Source: PLoS One. 2025 Sep 12;20(9):e0332068. doi: 10.1371/journal.pone.0332068 (PMC12431122; doi:10.1371/journal.pone.0332068)
Supplement: S1 Table — (DOCX) [file pone.0332068.s001.docx]

**S1 Table. Summary of statistics and analysis methods.**

| **Figure number** | **Comparing objects** | **Test used** | ***n* value** | **Statistical description** |
| --- | --- | --- | --- | --- |
| Figure 1A | Pre-transition (total), 2014 *vs* 2023 | Two-tailed unpaired t-test | 120 &120 | *T* = -30.829, *df* = 238, *P* < 0.001. |
|  | Transition (total), 2014 *vs* 2023 |  | 69 &641 | *T* = -16.581, *df* = 708, *P* < 0.001. |
| Figure 1B | Pre-transition (total) *vs* Transition (total) | Two-tailed unpaired t-test | 120 &69 (in 2014) | *T* = -12.351, *df* = 187, *P* < 0.001. |
|  |  |  | 120 &299 (in 2015) | *T* = -3.285, *df* = 417, *P* < 0.001. |
|  |  |  | 120 &350 (in 2016) | *T* = 4.524, *df* = 468, *P* < 0.001. |
|  |  |  | 120 &312 (in 2017) | *T* = 4.095, *df* = 430, *P* < 0.001. |
|  |  |  | 120 &598 (in 2018) | *T* = 3.447, *df* = 716, *P* < 0.001. |
|  |  |  | 120 &201 (in 2019) | *T* = 6.305, *df* = 319, *P* < 0.001. |
|  |  |  | 120 &395 (in 2020) | *T* = -8.806, *df* = 513, *P* < 0.001. |
|  |  |  | 120 &283 (in 2021) | *T* = 9.285, *df* = 401, *P* < 0.001. |
|  |  |  | 120 &572 (in 2022) | *T* = 9.658, *df* = 690, *P* < 0.001. |
|  |  |  | 120 &641 (in 2023) | *T* = 13.599, *df* = 759, *P* < 0.001. |
| Figure 1C | Pre-transition (total) *vs* Transition (basic) | Two-tailed unpaired t-test | 120 &69 (in 2014) | *T* = -10.964, *df* = 187, *P* < 0.001. |
|  |  |  | 120 &299 (in 2015) | *T* = -4.189, *df* = 417, *P* < 0.001. |
|  |  |  | 120 &350 (in 2016) | *T* = -9.038, *df* = 468, *P* < 0.001. |
|  |  |  | 120 &312 (in 2017) | *T* = -9.015, *df* = 430, *P* < 0.001. |
|  |  |  | 120 &598 (in 2018) | *T* = -6.922, *df* = 716, *P* < 0.001. |
|  |  |  | 120 &201 (in 2019) | *T* = -4.966, *df* = 319, *P* < 0.001. |
|  |  |  | 120 &395 (in 2020) | *T* = -6.082, *df* = 513, *P* < 0.001. |
|  |  |  | 120 &283 (in 2021) | *T* = -5.272, *df* = 401, *P* < 0.001. |
|  |  |  | 120 &572 (in 2022) | *T* = -4.65, *df* = 690, *P* < 0.001. |
|  |  |  | 120 & 641 (in 2023) | *T* = -4.569, *df* = 759, *P* < 0.001. |
| Figure 2A | Basic pension: men *vs* women in 2014 | Two-tailed unpaired t-test | 49 (men) & 20 (women) | *T* = 0.367, *df* = 67, *P* = 0.715. |
|  | Basic pension: men *vs* women in 2015 |  | 197 (men) & 102 (women) | *T* = -0.414, *df* = 67, *P* = 0.68. |
|  | Basic pension: men *vs* women in 2016 |  | 230 (men) & 120 (women) | *T* = -1.786, *df* = 348, *P* = 0.0749. |
|  | Basic pension: men *vs* women in 2017 |  | 208 (men) & 104 (women) | *T* = 1.502, *df* = 310, *P* = 0.134. |
|  | Basic pension: men *vs* women in 2018 |  | 271 (men) & 327 (women) | *T* = 9.613, *df* = 596, *P* < 0.001. |
|  | Basic pension: men *vs* women in 2019 |  | 83 (men) & 118 (women) | *T* = 11.357, *df* = 199, *P* < 0.001. |
|  | Basic pension: men *vs* women in 2020 |  | 273 (men) & 122 (women) | *T* = 8.993, *df* = 393, *P* < 0.001. |
|  | Basic pension: men *vs* women in 2021 |  | 175 (men) & 108 (women) | *T* = 5.172, *df* = 281, *P* < 0.001. |
|  | Basic pension: men *vs* women in 2022 |  | 444 (men) & 128 (women) | *T* = 3.321, *df* = 570, *P* < 0.001. |
|  | Basic pension: men *vs* women in 2023 |  | 397 (men) & 244 (women) | *T* = 2.416, *df* = 639, *P* = 0.016. |
| Figure 2B | Basic pension: men *vs* women at identical age | Two-tailed unpaired t-test | 83 (men, 2019) & 20 (women, 2014) | *T* = 9.788, *df* = 101, *P* < 0.001. |
|  |  |  | 273 (men, 2020) & 102 (women, 2015) | *T* = 16.412, *df* = 373, *P* < 0.001. |
|  |  |  | 175 (men, 2021) & 120 (women, 2016) | *T* = 10.733, *df* = 293, *P* < 0.001. |
|  |  |  | 444 (men, 2022) & 104 (women, 2017) | *T* = 12.529, *df* = 546, *P* < 0.001. |
|  |  |  | 397 (men, 2023) & 327 (women, 2018) | *T* = 28.889, *df* = 722, *P* < 0.001. |
| Figure 3A | Occupational annuities: men *vs* women in 2014 | Two-tailed unpaired t-test | 49 (men) & 20 (women) | *T* = -1.493, *df* = 67, *P* = 0.14. |
|  | Occupational annuities: men *vs* women in 2015 |  | 197 (men) & 102 (women) | *T* = 0.45, *df* = 297, *P* = 0.653. |
|  | Occupational annuities: men *vs* women in 2016 |  | 230 (men) & 120 (women) | *T* = -2.344, *df* = 348, *P* = 0.0196. |
|  | Occupational annuities: men *vs* women in 2017 |  | 208 (men) & 104 (women) | *T* = 5.651, *df* = 310, *P* < 0.001. |
|  | Occupational annuities: men *vs* women in 2018 |  | 271 (men) & 327 (women) | *T* = 12.997, *df* = 596, *P* < 0.001. |
|  | Occupational annuities: men *vs* women in 2019 |  | 83 (men) & 118 (women) | *T* = 11.807, *df* = 199, *P* < 0.001. |
|  | Occupational annuities: men *vs* women in 2020 |  | 273 (men) & 122 (women) | *T* = 11.646, *df* = 393, *P* < 0.001. |
|  | Occupational annuities: men *vs* women in 2021 |  | 175 (men) & 108 (women) | *T* = 7.188, *df* = 281, *P* < 0.001. |
|  | Occupational annuities: men *vs* women in 2022 |  | 444 (men) & 128 (women) | *T* = 3.198, *df* = 570, *P* < 0.00146. |
|  | Occupational annuities: men *vs* women in 2023 |  | 397 (men) & 244 (women) | *T* = 2.776, *df* = 639, *P* = 0.00566. |
| Figure 3B | Occupational annuities: men *vs* women at identical age | Two-tailed unpaired t-test | 83 (men, 2019) & 20 (women, 2014) | *T* = 17.681, *df* = 101, *P* < 0.001. |
|  |  |  | 273 (men, 2020) & 102 (women, 2015) | *T* = 28.268, *df* = 373, *P* < 0.001. |
|  |  |  | 175 (men, 2021) & 120 (women, 2016) | *T* = 25.851, *df* = 293, *P* < 0.001. |
|  |  |  | 444 (men, 2022) & 104 (women, 2017) | *T* = 20.834, *df* = 546, *P* < 0.001. |
|  |  |  | 397 (men, 2023) & 327 (women, 2018) | *T* = 45.176, *df* = 722, *P* < 0.001. |
| Figure 4A | Correlation between the standard basic pension and the basic pension of retirees in professional and technical staff | Pearson Correlation | 1860 (the standard basic pension) & 1860 (the basic pension) | CC = 0.963. |
|  | Correlation between the transitional pension and the basic pension of retirees in professional and technical staff |  | 1860 (the transitional pension) & 1860 (the basic pension) | CC = 0.839. |
|  | Correlation between the transitional pension and the basic pension of retirees in professional and technical staff |  | 1860 (the transitional pension) & 1860 (the basic pension) | CC = 0.851. |
|  | Correlation between the standard basic pension and the basic pension of retirees in management staff | Pearson Correlation | 1240 (the standard basic pension) & 1240 (the basic pension) | CC = 0.956. |
|  | Correlation between the transitional pension and the basic pension of retirees in management staff |  | 1240 (the transitional pension) & 1240 (the basic pension) | CC = 0.862. |
|  | Correlation between the transitional pension and the basic pension of retirees in management staff |  | 1240 (the transitional pension) & 1240 (the basic pension) | CC = 0.84. |
|  | Correlation between the standard basic pension and the basic pension of retirees in ground-skilled staff | Pearson Correlation | 1240 (the standard basic pension) & 620 (the basic pension) | CC = 0.936. |
|  | Correlation between the transitional pension and the basic pension of retirees in ground-skilled staff |  | 620 (the transitional pension) & 620 (the basic pension) | CC = 0.813. |
|  | Correlation between the transitional pension and the basic pension of retirees in ground-skilled staff |  | 620 (the transitional pension) & 620 (the basic pension) | CC = 0.926. |
| Figure 5A | Variations in occupational annuities between ground-skilled staff | One-way ANOVA | 116 (level Ⅰ) & 155 (level Ⅱ) & 203 (level Ⅲ) & 146 (level Ⅳ). | *F*_3, 619_ = 29.755, *P* < 0.001.  Level Ⅰ *vs* level Ⅳ, *P* < 0.001; Level Ⅱ *vs* level Ⅳ, *P* < 0.001; Level Ⅲ *vs* level Ⅳ, *P* = 0.684. |
| Figure 5A | Variations in occupational annuities between management staff | One-way ANOVA | 72 (level Ⅳ) & 402 (level Ⅴ) & 116 (level Ⅵ) & 297 (level Ⅶ) & 256 (level Ⅷ) & 72 (level Ⅸ). | *F*_5, 1239_ = 53.833, *P* < 0.001.  Level Ⅳ *vs* level Ⅸ, *P* < 0.001; Level Ⅴ *vs* level Ⅸ, *P* < 0.001; Level Ⅵ *vs* level Ⅸ, *P* = 0.684; Level Ⅶ *vs* level Ⅸ, *P* = 0.684; Level Ⅷ *vs* level Ⅸ, *P* = 0.415. |
| Figure 5A | Variations in occupational annuities between professional and technical staff | One-way ANOVA | 112 (level Ⅱ) & 326 (level Ⅲ) & 416 (level Ⅳ) & 233 (level Ⅴ) & 268 (level Ⅵ) & 182 (level Ⅶ) & 139 (level Ⅷ) & 102 (level Ⅸ) & 82 (level Ⅹ). | *F*_8, 1859_ = 53.833, *P* < 0.001.  Level Ⅱ *vs* level Ⅹ, *P* < 0.001; Level Ⅲ *vs* level Ⅹ, *P* < 0.001; Level Ⅳ *vs* level Ⅹ, *P* *P* < 0.001; Level Ⅴ *vs* level Ⅹ, *P* < 0.001; Level Ⅵ *vs* level Ⅹ, *P* < 0.001; Level Ⅶ *vs* level Ⅹ, *P* = 0.045; Level Ⅷ *vs* level Ⅹ, *P* = 0.384; Level Ⅸ *vs* level Ⅹ, *P* = 0.542. |
| Figure 5B | Variations in occupational annuities among ground-skilled staff, management staff, and professional and technical staff | One-way ANOVA | 620 (ground-skilled staff) & 1240 (management staff) & 1860 (professional and technical staff). | *F*_2, 3719_ = 326.857, *P* < 0.001.  Ground-skilled staff *vs* management staff, *P* < 0.001; ground-skilled staff vs professional and technical staff, *P* < 0.001. |
