# Supplementary material for: Pension levels of chinese institutions during the transition period: A case study of universities
Source: PLoS One. 2025 Sep 12;20(9):e0332068. doi: 10.1371/journal.pone.0332068 (PMC12431122; doi:10.1371/journal.pone.0332068)
Supplement: Attachment — Submitted filename: Lu et al__response to Reviewers.docx [file pone.0332068.s004.docx]

**Response to Reviewer # 1：**

Many thanks to the reviewer for his/her encouraging comments and valuable suggestions to our manuscript.

The following is our point-by-point reply to the issues raised by the reviewer:

**Comments to the Author**

1. Is the manuscript technically sound, and do the data support the conclusions?

The manuscript must describe a technically sound piece of scientific research with data that supports the conclusions. Experiments must have been conducted rigorously, with appropriate controls, replication, and sample sizes. The conclusions must be drawn appropriately based on the data presented.

Reviewer #1: Partly

2. Has the statistical analysis been performed appropriately and rigorously?

Reviewer #1: I Don't Know

3. Have the authors made all data underlying the findings in their manuscript fully available?

The [PLOS Data policy](http://track.editorialmanager.com/CL0/http:%2F%2Fwww.plosone.org%2Fstatic%2Fpolicies.action%23sharing/1/010f01966910e554-11233dd9-ccb8-4937-b6a0-3100c8523135-000000/0BylmKhs7LpZb6yRWexHGsnbamE3RuQQ2_DmzA94g_s=208) requires authors to make all data underlying the findings described in their manuscript fully available without restriction, with rare exception (please refer to the Data Availability Statement in the manuscript PDF file). The data should be provided as part of the manuscript or its supporting information, or deposited to a public repository. For example, in addition to summary statistics, the data points behind means, medians and variance measures should be available. If there are restrictions on publicly sharing data—e.g. participant privacy or use of data from a third party—those must be specified.

Reviewer #1: No

4. Is the manuscript presented in an intelligible fashion and written in standard English?

PLOS ONE does not copyedit accepted manuscripts, so the language in submitted articles must be clear, correct, and unambiguous. Any typographical or grammatical errors should be corrected at revision, so please note any specific errors here.

Reviewer #1: Yes

5. Review Comments to the Author

Please use the space provided to explain your answers to the questions above. You may also include additional comments for the author, including concerns about dual publication, research ethics, or publication ethics. (Please upload your review as an attachment if it exceeds 20,000 characters)

Reviewer #1: The article addresses a globally relevant issue: population aging and its implications for pension systems. In particular, the authors focus on the Chinese pension system, analyzing the effects of a reform initiated in 2014 and structured over a ten-year transitional period.

In my opinion, although the topic is relevant and the study presents some interesting aspects, the manuscript requires a major revision before it can be considered for publication.

Among the strengths of the paper, I acknowledge:

• the clear relevance of the subject;
• the size of the dataset, which includes 1,200 retirees from before the transition and 3,270 from during the transition period.
However, several weaknesses compromise the overall quality and clarity of the manuscript. In particular:

1. Formatting issues with mathematical content

The mathematical formulas are presented inline (e.g., page 15), rather than in a proper equation environment. This makes them difficult to read. Furthermore, many symbols are introduced after the formulas, reducing their immediate comprehensibility. The mathematical notation should be cleaned up, with clearly defined variables and standalone, numbered equations where appropriate.

**Response:** According to the Reviewer’s suggestion, we have employed mathematical formulas to illustrate the calculation methods for pre-transition and transition pensions, with each component clearly defined and elucidated in relation to the formulas (Page 6, Lines 138-139, and Pages 7-8 in the revised manuscript). We hope that this approach will facilitate a more accessible reading experience for the audience.

2. Lack of explanation for key techniques

Several methods and indices are used without any introduction or justification. These should be properly explained, especially for non-expert readers.

**Response:** According to the Reviewer’s suggestion, we have included the statistical methods utilized in the figure legends. Additionally, we have incorporated statistical tables into the manuscript that provide detailed information on the tests employed, sample size (*n*), and statistical descriptions (S1 Table of the revised manuscript). Furthermore, we have revised what was originally Table 2 to Figure 4 to enhance the clarity and intuitiveness of the results (Figure 4 of the revised manuscript).

3. Impression of a rushed submission

The writing gives the impression that the paper was submitted prematurely. A thorough revision is necessary to improve both technical clarity and narrative cohesion. Moreover, clicking on the link to the dataset opens a page in Chinese. It would be better to upload the data to a public repository with an explanation in English, so that access is understandable internationally.

**Response:** According to the Reviewer’s suggestion, we have made substantial revisions to the manuscript, which include the incorporation of formulas for pension calculation and a comprehensive definition of each component within the pension framework (Page 6, Lines 138-139, and Pages 7-8 in the revised manuscript). Secondly, we provide a detailed clarification of the statistical methods employed in this study within the methods section and accompanying figure legends. Thirdly, in S1 Table, we present an exhaustive description of the statistics utilized in each figure throughout the manuscript. This includes information on comparison objects, statistical methods applied, sample sizes, and corresponding statistical results (S1 Table of the revised manuscript). Finally, due to the presence of sensitive information related to individual income, we have revised the data availability statement (Page 26, Lines 617-627 in the revised manuscript).

To conclude, I believe the study addresses an important and interesting question, and the data used are valuable. However, the authors should revise the manuscript with greater care and avoid assuming too much background knowledge on the part of the reader.

6. PLOS authors have the option to publish the peer review history of their article ([what does this mean?](https://track.editorialmanager.com/CL0/https:%2F%2Fjournals.plos.org%2Fplosone%2Fs%2Feditorial-and-peer-review-process%23loc-peer-review-history/1/010f01966910e554-11233dd9-ccb8-4937-b6a0-3100c8523135-000000/Q83_W9HzFbAaUK-IM2Cn76FVAsRRulWbzzHAGq1EslI=208)). If published, this will include your full peer review and any attached files.

If you choose “no”, your identity will remain anonymous but your review may still be made public.

**Do you want your identity to be public for this peer review?** For information about this choice, including consent withdrawal, please see our [Privacy Policy](https://track.editorialmanager.com/CL0/https:%2F%2Fwww.plos.org%2Fprivacy-policy/1/010f01966910e554-11233dd9-ccb8-4937-b6a0-3100c8523135-000000/M_rZz-cvEiFmRkXlm7eKKkvh7jOrxyIxcBW4_9Ivve8=208).

Reviewer #1: No

[NOTE: If reviewer comments were submitted as an attachment file, they will be attached to this email and accessible via the submission site. Please log into your account, locate the manuscript record, and check for the action link "View Attachments". If this link does not appear, there are no attachment files.]

While revising your submission, please upload your figure files to the Preflight Analysis and Conversion Engine (PACE) digital diagnostic tool, [https://pacev2.apexcovantage.com/](https://track.editorialmanager.com/CL0/https:%2F%2Fpacev2.apexcovantage.com%2F/1/010f01966910e554-11233dd9-ccb8-4937-b6a0-3100c8523135-000000/etCKDx1UIr_IgxqeAb_nk2jwfgzSRKl3xyF0JZgzVBU=208). PACE helps ensure that figures meet PLOS requirements. To use PACE, you must first register as a user. Registration is free. Then, login and navigate to the UPLOAD tab, where you will find detailed instructions on how to use the tool. If you encounter any issues or have any questions when using PACE, please email PLOS at [figures@plos.org](mailto:figures@plos.org). Please note that Supporting Information files do not need this step.
