# Supplementary material for: Pension levels of chinese institutions during the transition period: A case study of universities
Source: PLoS One. 2025 Sep 12;20(9):e0332068. doi: 10.1371/journal.pone.0332068 (PMC12431122; doi:10.1371/journal.pone.0332068)
Supplement: Attachment — Submitted filename: Lu et al__response to Editor and Reviewers.docx [file pone.0332068.s005.docx]

**Response to Editor：**

Many thanks to the editor for her encouraging comments and valuable suggestions to our manuscript.

The following is our reply to the point raised by the academic editor:

Based on my own assessment, I agree with the points raised by the referee. In addition, as I mentioned in my previous email, I still do not see a stronger connection with the relevant theoretical literature, particularly in relation to the main findings. I therefore kindly ask you to address this aspect in your revised manuscript.

**Response:** According to the Editor’s suggestion, we have incorporated a discussion regarding the relationship between the primary findings of this manuscript and the relevant theoretical literature in the discussion section (Page 21, Lines 490-513 in the revised manuscript).

**Response to Reviewer # 1：**

Many thanks to the reviewer for his encouraging comments and valuable suggestions to our manuscript.

The following is our reply to the points raised by the reviewer:

**Comments to the Author**

1. Have the authors made all data underlying the findings in their manuscript fully available?

The PLOS Data policy requires authors to make all data underlying the findings described in their manuscript fully available without restriction, with rare exception (please refer to the Data Availability Statement in the manuscript PDF file). The data should be provided as part of the manuscript or its supporting information, or deposited to a public repository. For example, in addition to summary statistics, the data points behind means, medians and variance measures should be available. If there are restrictions on publicly sharing data—e.g. participant privacy or use of data from a third party—those must be specified.

Reviewer #1: No

**Response:** In accordance with the submission guidelines, we have included a "Data Availability Statement" at the end of the manuscript, which has been approved by the editorial manager.

2. Before proceeding with publication, I suggest making the following final revisions:

- Line 168: Is the % sign necessary?

**Response:** Thanks very much for the Reviewer’s suggestion, we have carefully examined the formula and determined that the percentage sign is indeed necessary.

- Lines 168–169: Move the entire formula for A_PI to line 169.

**Response:** According to the Reviewer’s suggestion, we have relocated the entire formula for A_PI to the subsequent line (Page 8, Line 170 in the revised manuscript).

- Line 178: The subscripts n and n − 1 are too large. If the article was written in Word, I suggest using the equation environment or at least reducing the size of the subscripts and ensuring consistency throughout the article (e.g., on line 180). If the article was written in LaTeX, I suggest using something like $x_n$ and then making adjustments wherever needed.

**Response:** Thanks very much for the Reviewer’s suggestion, we have reduced the size of the subscripts and ensured consistency throughout the manuscript (Page 8, Lines 179-181 in the revised manuscript).

- Line 202: For consistency with the rest of the article, "presented in S1 Table" should be changed to "presented in (Table S1)", with the parentheses and their content in bold.

**Response:** According to the Reviewer’s suggestion, we have revised the representation of Table S1 (Page 8, Line 203 in the revised manuscript).
